# Lockdowns lose one third of their impact on mobility in a month

**DOI:** 10.1038/s41598-021-02133-1

**Published:** 2021-11-22

**Authors:** Yogesh V. Joshi, Andres Musalem

**Affiliations:** 1 Robert H. Smith School of Business, University of Maryland, College Park, 20742 USA; 2 Department of Industrial Engineering, University of Chile, Santiago, Chile; 3 Instituto Sistemas Complejos de Ingeniería, Santiago, Chile

**Keywords:** Epidemiology, Health policy

## Abstract

As the novel coronavirus (COVID‐19) pandemic spread across the world over the past year, many countries imposed lockdowns in the form of stay at home requirements on their citizens to mitigate its spread. We analyze mobility data from 93 countries implementing lockdowns to investigate their immediate impact on mobility and the subsequent evolution of mobility. We find that at the start of a lockdown, median mobility is reduced to 36% below the baseline, and by another 18% in the subsequent 2 weeks. 70 countries had lockdowns lasting beyond 4 weeks and showed a significant reduction in mobility compared to that prior to the lockdown. Mobility was at its minimum 18 days into the lockdown for the median country. Comparing this minimum mobility to the average mobility 2 weeks before the lockdown, we observe a median reduction of 50 percentage points, evidencing that lockdowns reduce mobility. For 59 of these 70 countries, lockdowns lasted at least 4 weeks after reaching minimum mobility and most observed a significant rebound in mobility during the lockdown period. For the median country, 30.1% of the mobility reduction achieved is lost within 4 weeks, and lockdowns lose all their impact on mobility in 112.1 days. Overall, our findings show that while lockdowns significantly reduce mobility, this impact is also subject to fatigue as the lockdown period extends longer. The magnitude of mobility reductions achieved and fatigues reported in this research can help policy makers anticipate the likely impact of their lockdown policies.

## Introduction

The United States recorded its first confirmed case of the novel coronavirus disease (COVID-19) in late January, 2020 in the state of Washington^1^. By the end of January, thousands of cases were observed in China and cases appeared to be spreading globally, leading the World Health Organization (WHO) to declare COVID-19 to be a Public Health Emergency of International Concern^2^. By March 2020, COVID-19 had spread to over a hundred countries with over a hundred thousand cases and more than four thousand dead, further leading the WHO to declare it a global “pandemic”^3^.

As COVID-19 spread globally, governments in many countries started instituting formal policies with the aim of mitigating the potential outbreak and loss of life within their individual jurisdictions. Starting with travel advisories and travel restrictions, the policies quickly escalated to more severe actions such as stay at home requirements (SHR) which we refer to as lockdowns. The intent of imposing lockdowns was to restrict and reduce the movement of citizens, which in turn would lead to lower contact among people, and hence to lower cases and lesser loss of life^4,5^. Indeed, scholars have analyzed the impact of mobility on the spread of the disease and shown that a reduction in mobility can slow the spread^6–9^.

At the same time, many have questioned the appropriateness of governments imposing lockdowns and the general efficacy of lockdowns and quarantines^10^. The editorial board of the Wall Street Journal publicly opined “These shutdowns are extraordinary and have costs, not least the harm to small business owners. Americans may simply decide to ignore the orders after a time. Absent a more thorough explanation of costs and benefits, we doubt these extreme measures will be sustainable for long as the public begins to chafe at the limits and sees the economic consequences”^11^. More generally, research shows that government policy, the alignment of public interests, and compliance interact closely^12,13^. In this research, we specifically focus our attention on the broader issue of whether and to what extent have lockdowns had an impact on mobility.

A casual observation of the policies adopted by governments around the world suggests that the severity of the rules as well as the extent of their enforcement has varied significantly across countries. At the same time, the impact of these lockdown policies on subsequent mobility has also been varied. Motivated by these observations, in this work we investigate the following research questions: (i) What has been the impact of lockdowns on mobility? (ii) Is this impact long-lasting, at least during the time when lockdowns are in effect? (iii) If there is variation in this long-term impact across countries, what mobility patterns and country characteristics explain these differences?

Our work contributes to a growing body of research analyzing the impact of government policies on factors such as mobility^14,15^. Prior research has found that government policies as well as pandemic severity impacts social distancing that is practiced within communities, and that less social distancing is practiced after observing local mitigation^16^. Researchers have also shown that social distancing and lower population density may be associated with decreased spread of the coronavirus^17^. Other work has shown that risk attitudes can be a critical factor in predicting mobility reduction, and that regions with more risk averse attitudes may be more likely to change behavior in a pandemic^18^. Compliance with mobility restrictions have also been shown to be associated with social connections and partisan beliefs^19,20^. We focus on the impact of lockdowns on mobility and characterize the extent and dynamics of the reduction in mobility achieved by imposing lockdowns in countries across the world. We contribute to existing research by quantifying the magnitudes of the initial impact of lockdowns on mobility, as well as the magnitudes of the subsequent evolution of mobility levels during the lockdown. Knowledge of these magnitudes can help policy makers better anticipate the impact of their actions.

## Empirical approach

To study the impact of lockdowns on mobility we analyze policy and mobility data for 116 countries. The outcome variable in our analysis is the daily workplace mobility data relative to a baseline level of mobility as reported by Google. This data is measured by Google using mobile devices of users who have opted in to share the Location History for their Google accounts. The data shows how user visits and length of stay at different places change compared to a baseline value for that day of the week. Specifically, it measures the percentage change in visits to places of work compared to that baseline. The baseline is the median value, for the corresponding day of the week, during the 5-week period Jan 3–Feb 6, 2020. We analyze the data using the R^21^ and RStudio^22^ software (see Supplementary Information 1 and 2).

For each country, we identify the first time that a general requirement to stay at home (i.e., a lockdown) is imposed on its citizens and its duration. Using the metric of mobility at workplaces, we compute a baseline level of mobility prior to the start of the lockdown for that country to understand mobility levels before restrictions went into effect. Using regression models that control for day-of-the-week effects we characterize the evolution of mobility levels throughout the duration of a lockdown. Via further regression analyses, we assess whether differences across countries in mobility responses to lockdowns can be explained by country characteristics such as demographics and socioeconomic indicators.

### Mobility levels are lower during lockdowns

**Figure 1:**
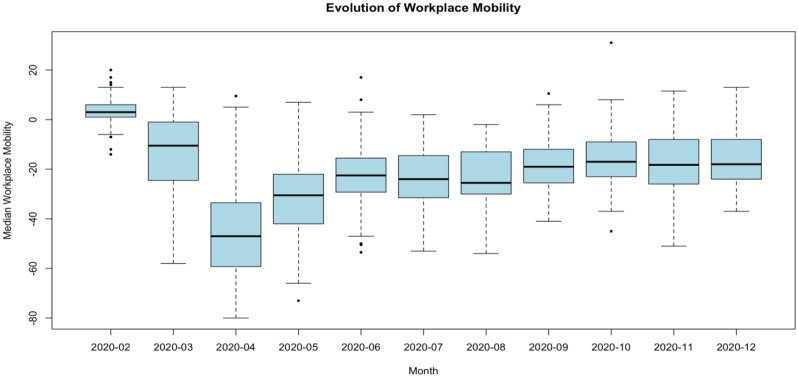
Evolution of workplace mobility. Boxplot of the average workplace mobility for all countries by month (n = 116 countries). The solid line in the box indicates the median, with boxes at the interquartile range. Whiskers are either at 1.5 × (interquartile range) outside the box, or at the extreme value.

To understand mobility patterns over the past year, regardless of policy or other interventions, we first compute the median mobility by month in each country for all 116 countries under study and generate a boxplot of these medians by month (Fig. 1). During the initial phase, we observe a global median decline in mobility of 10.5 and 47.0 percentage points in March and April 2020 respectively, compared to the baseline reference levels of January–February 2020. This is followed by a pattern of gradual and partial recovery that extends through June 2020, with mobility levels remaining relatively stable thereafter.

**Figure 2:**
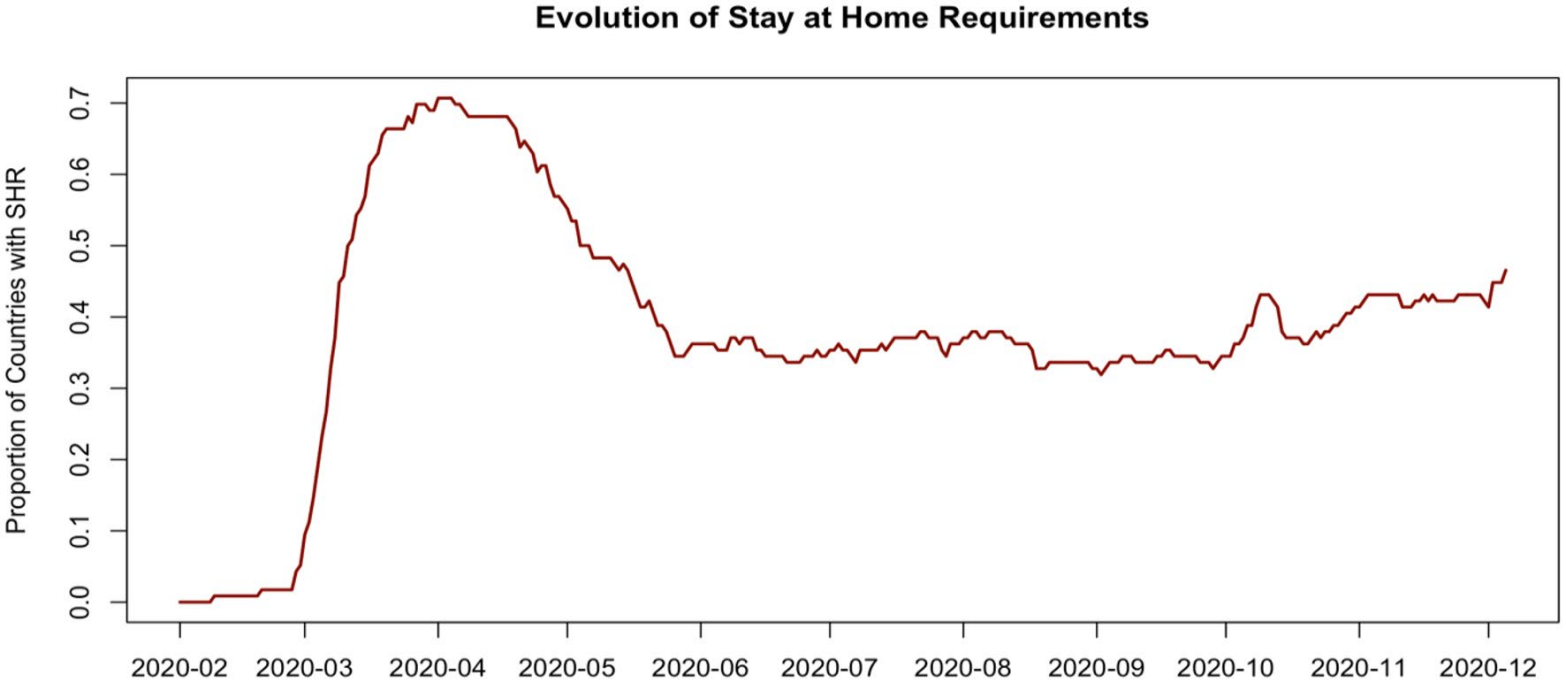
The proportion of countries with a lockdown. The daily fraction of countries that have an active stay-at-home order for its general population (n = 116 countries).

Next, we visualize the implementation of lockdown policies across the world as measured by the proportion of countries in our sample that are under lockdown on any given day (Fig. 2). Interestingly, the mobility decline and recovery observed in Fig. 1 coincides with the implementation and subsequent relaxation of lockdowns observed in Fig. 2. The highest proportion of countries requiring their citizens to stay at home is observed on April 15th, 2020 (70.7%). This proportion stayed at high levels during March to May 2020, and then decreased to lower levels in June 2020, and stayed low for a long time thereafter.

### Lockdowns have a strong initial impact and then fatigue sets in

**Figure 3:**
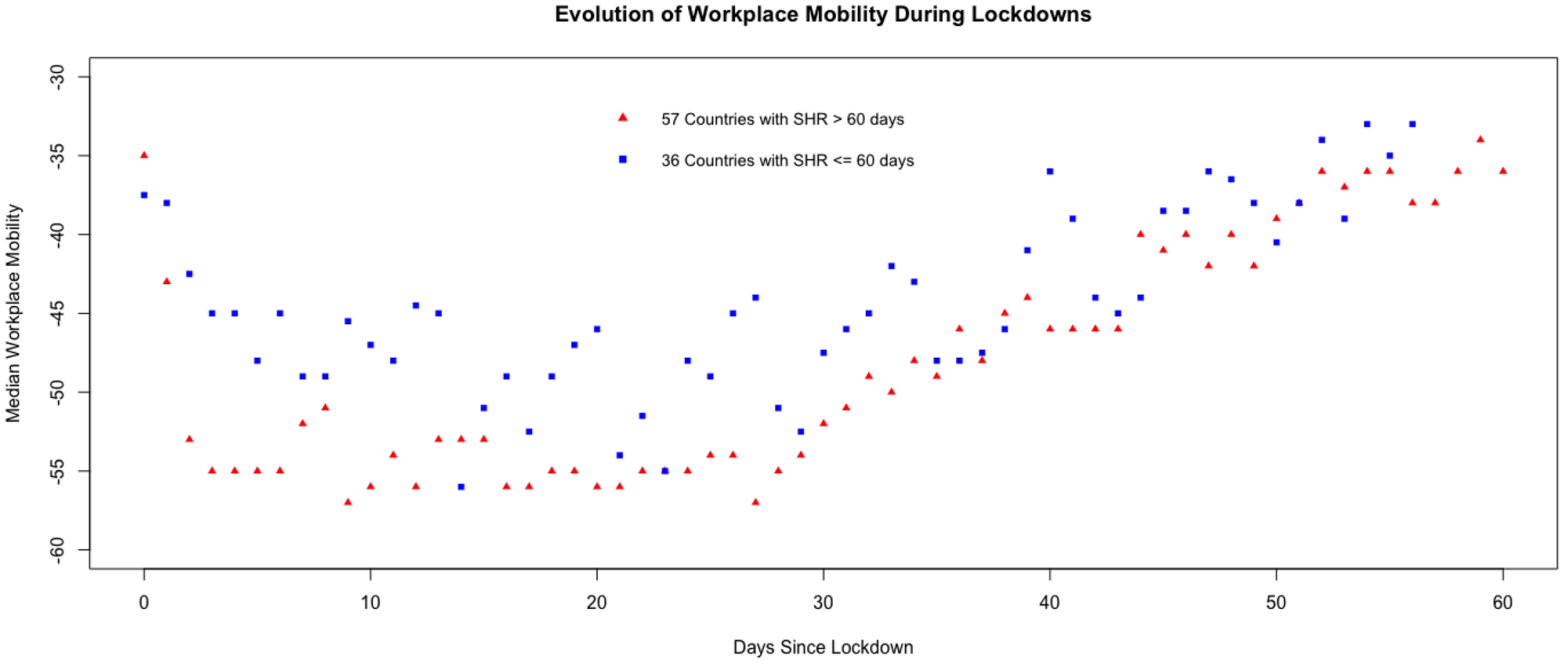
Evolution of workplace mobility during lockdowns. For each country (n = 93), we identify the date when the first lockdown (*SHR* stay at home requirement) was imposed over its general population. We separate countries by whether the imposed lockdown ≤ 60 days (blue squares) or > 60 days (red triangles). During the lockdown period, for each day since the start of the lockdown we determine the median workplace mobility across countries within each set for that day.

While at a macro level the prior two figures suggest that lockdowns impact mobility, a closer look at the evolution of mobility during lockdowns for each country reveals a more nuanced story. Figure 3 shows that imposing lockdowns leads to a strong initial reduction in mobility, but this is followed by a wear out period suggesting that lockdown fatigue is setting in. Across 93 countries that imposed a lockdown, the median mobility reduced to 36% below the baseline at the start of a lockdown, and then by another 18% two weeks later. Nevertheless, this initial impact exhibits wear out, as mobility gradually rises by approximately 3 percentage points with each additional week that the lockdown stays in effect. After 52 days of lockdown, the median mobility levels rise to 35% below the baseline, effectively erasing all impact of the lockdown, even though the lockdown continues to stay in effect.

As one may expect, the duration for which lockdowns are imposed varies across countries. Hence, it could be hypothesized that the fatigue observed in Fig. 3 might arise due to a selection effect if countries where lockdowns have a lower impact lift their lockdowns earlier. To analyze whether this is the case, in Fig. 3, we separately plot the median mobility across countries that have lockdowns lasting more than 60 days (red triangles) and the rest with lockdowns 60 days or shorter (blue squares). Countries with longer lockdowns do exhibit a greater reduction in mobility. These differences could arise, for example, if policy makers set the lockdown duration after assessing their early impact on mobility. In other words, compliance, along with metrics such as observed new cases, hospitalizations, case severity and deaths, could play an important role. Nevertheless, Fig. 3 reveals a similar pattern of lockdown fatigue for countries with shorter and longer duration of lockdowns.

### Lockdowns significantly reduce mobility

To better understand the dynamic impact of imposing a lockdown on mobility at the country level, we focus on 79 countries where lockdowns lasted at least 4 weeks. Out of these 79 countries, 70 exhibit a significant mobility reduction (p < 0.01) in the first two weeks of the lockdown compared to the 2 weeks before the lockdown. The median value of this reduction is 40 percentage points. The mobility data exhibits strong seasonality patterns based on the day of the week, hence we compute a 7 day moving average of mobility level during the lockdown to better understand lockdown dynamics. Based on this metric, the minimum level of mobility is achieved 18.0 days into the lockdown for the median country. Comparing this minimum mobility to the average mobility 2 weeks before the lockdown, we observe a median reduction of 50 percentage points. These observations indicate that lockdowns act to reduce mobility.

### 30% of the impact of a lockdown is lost in 4 weeks

For 67 of the 70 countries mentioned above (i.e., 96%), we observe a significant rebound in mobility during the lockdown period. Among them, for the median country, we find that 30.1% of the mobility reduction achieved from imposing a lockdown is lost within 4 weeks after reaching this minimum level. We then specify regression models to explain the observed daily mobility level within a country as a function of the days since the lockdown was imposed, after controlling for day of the week effects (additional details provided under “Materials and methods” section). For these countries, we used the fitted models to estimate the number of days it would take a lockdown to reach the average mobility levels observed during the 2 weeks before the start of the lockdown. This calculation reveals that lockdowns would lose all their impact in 112.1 days for the median country.

### Lower prior mobility levels are associated with greater lockdown fatigue

An important objective of this analysis is to better understand the factors associated with lockdown fatigue. To do so, we begin by studying the mobility before the start of a lockdown. The reason for this is to assess how a country’s population may respond to a pandemic in the absence of a lockdown policy.

If the mobility level prior to a lockdown was already low, this suggests that the country’s population had already restricted its mobility without the imposition of a lockdown policy. Consequently, after its imposition, this population may experience more fatigue due to their prior mobility reduction. Hence their compliance may wear out faster, and this may manifest as a stronger rebound in mobility during the lockdown.

**Figure 4:**
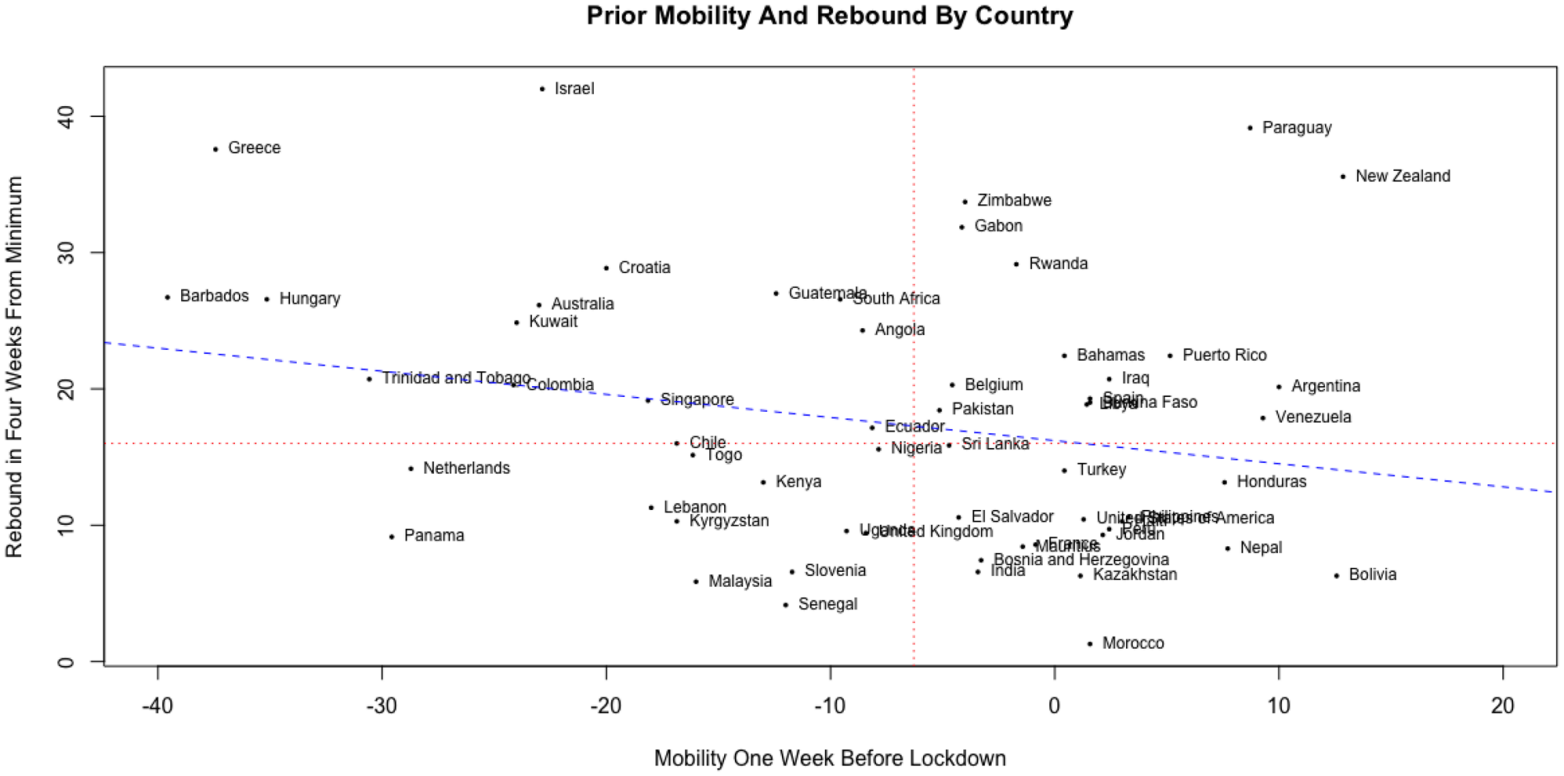
Prior mobility and rebound across n = 59 countries. The horizontal axis represents the average mobility level 1 week before the start of the lockdown. The vertical axis represents the rebound in mobility in 4 weeks starting from the day when minimum mobility levels were achieved during the lockdown.

To analyze whether this is the case, consider the country level data plotted in Fig. 4 for 59 countries with lockdowns lasting at least 4 weeks after reaching the minimum mobility level. The horizontal axis represents the average mobility level 1 week prior to the start of the lockdown. The vertical axis represents the mobility rebound, measured as the mobility gain in 4 weeks after reaching the minimum level during the lockdown. We observe a marginally significantly negative correlation between the two metrics (correlation =  − 0.23, p-value = 0.08), which suggests that countries that achieved lower mobility levels prior to the start of a lockdown exhibited a greater rebound in mobility.

### Greater mobility reductions are also associated with greater lockdown fatigue

A second factor that may impact lockdown fatigue is the population’s initial response to the imposition of the lockdown. We measure this response in terms of the reduction in mobility achieved between the start of a lockdown and the date when the minimum mobility level is reached.

The sign of the relationship between the initial response and lockdown fatigue is not a priori evident. On the one hand, in countries with greater mobility drops, mobility may remain low if the cost of compliance with the lockdown is low, if the penalty for not complying is high and strictly enforced, or if the general population has an intrinsic and sustained motivation to comply with government mandates. On the other hand, certain segments of the population may lack resources to be self-sufficient when staying at home, experience depletion of self-control, or question the effectiveness of prolonged lockdowns. If penalties are not too high or not strictly enforced, these factors could lead to a rebound in mobility after a strong initial mobility reduction.

**Figure 5:**
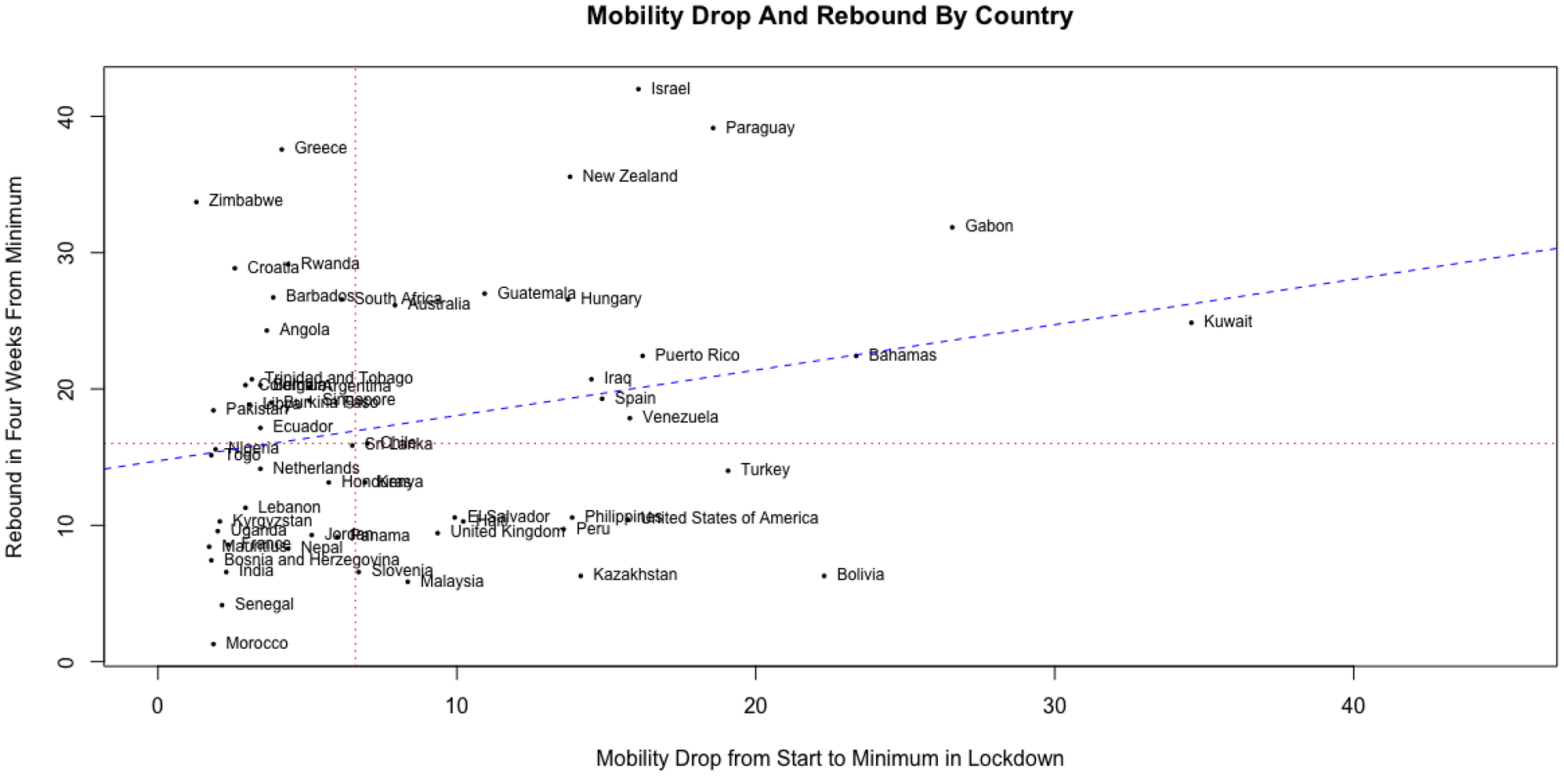
Mobility drop and rebound across n = 59 countries. The horizontal axis represents the mobility drop from the start of the lockdown until reaching the minimum mobility level during the lockdown. The vertical axis represents the rebound in mobility in 4 weeks from the day when minimum mobility levels were achieved during the lockdown.

To analyze this relationship, consider the data plotted in Fig. 5. The horizontal axis represents the mobility drop from one week prior to the lockdown to the minimum level of mobility observed during the lockdown. The vertical axis represents mobility rebound, similar to Fig. 4. We observe a significantly positive correlation between the two metrics (correlation = 0.25, p-value = 0.05), which suggests that countries that achieved a greater reduction in mobility from the start of a lockdown exhibited a greater rebound in mobility.

### Country characteristics and mobility dynamics

We observe substantial variation in mobility dynamics during the lockdown across countries. We next explore whether country characteristics help explain some of this variation. We focus on three mobility metrics: (i) mobility levels observed prior to the lockdown; (ii) the reduction in mobility achieved between the 2 weeks prior to the lockdown and the date when the minimum mobility is achieved; and (iii) the rebound in the mobility levels observed 4 weeks after reaching the minimum mobility levels during the lockdown. We conduct regression analyses with each of these three metrics as a dependent variable and country characteristics as predictors (please refer to the methods and materials section for a list of the country characteristics used for each regression).

First, in terms of the analysis of mobility levels prior to the lockdown, we find that both the policies adopted by a country before a lockdown as well as its education levels are significant predictors for this metric. In particular, we obtain a significantly negative impact of the stringency index observed prior to the lockdown and the mean years of schooling of a country.

Second, considering the reduction of mobility achieved between the start of a lockdown until reaching the minimum mobility level, once again the policies implemented prior to the start of the lockdown significantly predict this mobility reduction. More specifically, more stringent policies prior to the lockdown are associated with a weaker drop in mobility. In addition, countries with populations enjoying a greater life expectancy are associated with stronger reductions in mobility.

Third, regarding the mobility rebound realized four weeks after reaching the minimum level, both the mobility levels observed prior to the start of the lockdown and the mobility reduction achieved during lockdown are significant predictors with opposite directions. On the one hand, greater mobility levels prior to the start of a lockdown are associated with a weaker mobility rebound. On the other hand, a greater mobility reduction achieved during the lockdown is associated with a stronger mobility rebound.

## Discussion

This work analyzes the impact of lockdowns on mobility at its onset as well as over a longer horizon during the time it is in effect. It also investigates the variation in mobility patterns across countries and whether country characteristics explain the observed variation. Below we summarize our main conclusions and relate them to prior work, and end by discussing the limitations of this work and ideas for future research.

### Conclusions

Overall, our global analysis suggests that lockdowns work, in that imposing a lockdown is associated with a significant reduction in the observed levels of mobility for most countries in our data. These findings are consistent with prior research at the state level in the US which also reports a reduction in mobility when states impose stay-at-home orders^14^. In our work, we also observe and quantify a global pattern of lockdown fatigue, as mobility levels slowly start rising the longer a lockdown stays in effect. A direct implication of this result is that lockdown compliance might need to be reinforced by governments when lockdowns remain in effect for long periods of time. We now discuss our findings and their implications in more detail.

The primary goal of implementing strict lockdowns is to reduce infections among the population. Prior research has shown that reducing mobility helps reduce infections^9^. Coupled with the findings from our global analysis that strict lockdown policies are associated with a significant reduction in mobility, one can infer that lockdowns can be effective at achieving their primary goal.

Previous simulations have suggested that lockdowns should be imposed for a substantial duration, such as 60 days, to contain the spread of the pandemic^15^. In practice, we observe that it takes time for an imposed lockdown to have an impact on mobility levels. We observe that mobility levels were at their minimum at 18 days into the lockdown for the median country. We also find that a prolonged use of these strict policies may lead to lockdown fatigue. Most countries, where lockdowns lasted for at least 4 weeks after reaching minimum mobility levels, observed a significant rebound in mobility. For the median country, 30.1% of the mobility reduction achieved is lost within 4 weeks, and lockdowns lose all their impact on mobility after 112.1 days.

Our results also show that a strict lockdown policy that leads to a greater reduction in mobility is associated with stronger fatigue. A similar association is observed between lockdown fatigue and the mobility levels prior to the start of the lockdown. Countries that had already achieved lower levels of mobility prior to a lockdown exhibit greater mobility rebounds during their lockdown periods. Interestingly, we find that lower mobility prior to a lockdown is observed for countries with greater education levels. Countries with more risk averse attitudes have been reported to change behavior even before a strict lockdown policy is imposed^18^. Our analysis shows that these countries are likely to experience greater lockdown fatigue.

The impact of lockdowns is also associated with other country characteristics such as the life expectancy enjoyed by the population in each country. Our global analysis finds that countries with greater life expectancy show stronger mobility reductions during a lockdown. The opposite is observed for countries that had more strict policies prior to the implementation of a lockdown.

### Limitations and future research

Our analysis has several limitations, which can serve as motivation for future research in this area. First, we do not intend to make any causal claims; our findings are based on summarizing and regressing available data across sources to study the interplay between government policy and mobility. Second, our inferences of mobility are based on workplace mobility data. While other mobility types have a strong positive correlation with this metric, future research could further investigate the impact of lockdowns on other types of mobility. Third, in this research we do not incorporate the explicit impact of case data on mobility. Future research can investigate whether observing actual cases within their communities may have a further impact on people’s decisions regarding mobility. Finally, this work focuses on the impact of first lockdowns on mobility. It may be interesting in future research to study the compliance and mobility response for subsequent lockdowns imposed within the same countries.

Our findings above may inform policy makers on the nature of response they may expect when implementing lockdowns and incorporate these observations for more effective planning purposes.

## Materials and methods

### Data

The data used in this research was compiled from three sources:

1. *Google COVID-19 Community Mobility Reports* This dataset was accessed on March 2nd 2021, and provides daily mobility data, relative to a baseline level of mobility as measured during a reference period of January 3 to February 6, 2020. Mobility measures were constructed by Google using mobile devices of users who have opted in to Location History for their Google accounts. There are six distinct mobility measures: workplace, retail & recreation, grocery & pharmacy, parks, transit stations and residential. The first five are highly and positively correlated with each other, while the sixth is negatively correlated with the first five measures. Of these measures, workplace mobility exhibits the fewest missing values, hence we use this measure as our metric for mobility. This metric was available starting February 15, 2020 through February 26, 2021.
  Additional details regarding the outcome variable are available at https://github.com/GoogleCloudPlatform/covid-19-open-data/blob/main/docs/table-mobility.md.
  We focus on data aggregated at the country level, giving us 246 distinct entities labeled as countries, although some of these are territories such as Puerto Rico. For ease of exposition, we refer to these 246 territories as countries. Of these 246, we exclude countries that have missing values for workplace mobility, leaving us with 116 countries.
  For each country, the data also includes characteristics such as population and geographical area.
2. Oxford COVID-19 Government Response Tracker^23^: This dataset was accessed on March 2nd 2021, and provided daily policy data on when each country (or state in the case of US) began requiring its citizens to stay at home. Government policy data regarding stay at home requirements (SHR) is provided on an ordinal scale, where 0 is *no measures* are in place, 1 is government *recommending* not leaving the house, 2 is the government *requiring* not to leave the house with exceptions for daily exercise, grocery shopping, and ‘essential’ trips; and 3 is the government *requiring* not leaving house with minimal exceptions (e.g., allowed to leave once a week, or only one person can leave at a time, etc.). We classify SHR levels 2 and 3 as being under a lockdown, since both these levels *require* a citizen to stay at home. A second variable in this dataset indicates whether SHRs are targeted to specific geographic regions (e.g., certain counties or regions), as opposed to being applied to the general population across the country. The final variable of interest from this dataset is the Stringency Index, which provides a composite score in the range of 0–100 (higher being more stringent) based on nine distinct policies related to school and workplace closings, cancellation of public events and public transport, restrictions on gatherings and on internal movements, on international travel, and whether the government is running public information campaigns.
3. The United Nations Development Project (UNDP) data: This dataset was accessed on March 9th, 2021, and contains country level data on gross national income per capita, life expectancy at birth in years, expected years of schooling, mean years of schooling. When merging this data set with the previous ones, we manually checked for matching country names where needed.

### Methods

The analysis for each country begins by identifying the first date when a lockdown is applied to the general population. Hence, we find the earliest date for which the variable C6_Stay at home requirements is at a level of 2 or 3 and the C6_Flag variable equals 1. The end and hence the duration of this first stay-at-home requirement is determined by finding the first date after the beginning of the requirement for which either C6_Stay at home requirements is at a level of 0 or 1 or the C6_Flag equals 0. Let the *s*_*i*_ and *e*_*i*_ denote the start and end of the lockdown for country *i*. Both variables are measured from the beginning of the 2020 calendar year. For example, a lockdown starting on March 15th 2020 and ending on March 29th 2020 would yield values of *s*_*i*_ and *e*_*i*_ equal to 74 and 88, respectively. We consider all periods *t* between the beginning and end of the lockdown for each country and we define the following six dummy variables Monday_*t*_,…, Saturday_*t*_ , which are equal to 1 if the period corresponds to a Monday,…, Saturday, respectively, and otherwise these variables are equal to 0. With these definitions, for each country *i* with a lockdown period of at least 3 weeks we first estimate the following linear model of workplace mobility m_*it*_ for country *i* in period *t*:

$${\text{m}}_{it} = \upalpha_{{\text{i}}} + \, \upbeta_{{\text{i}}} \left( {t - s_{i} } \right) \, + \, \uppi_{{1}} \,{\text{Monday}}_{t} + \, \uppi_{{2}} \,{\text{Tuesday}}_{t} + \, \uppi_{{3}} \,{\text{Wednesday}}_{t} + \, \uppi_{{4}} \,{\text{Thursday}}_{t} + \, \uppi_{{5}} \,{\text{Friday}}_{t} + \, \uppi_{{6}} \,{\text{Saturday}}_{t} + {\text{ e}}_{it} ,{\text{ where }}t\,{\text{in }}s_{i} ,\ldots,e_{i} .$$

In this linear model, α_i_ measures the expected mobility at the beginning of the lockdown, while β_i_ measures the change in mobility as the lockdown for country *i* is extended for an additional day. Hence, the latter coefficient yields a measure of fatigue in terms of the daily wear out for country i. Finally, e_*it*_ represents the error term of the model for country *i* in period *t*.

We then estimate a more flexible version of this model allowing for a change in the trend slope where:

$$\upbeta_{i} \left[ {\text{t}} \right] = \upbeta_{i0} + \beta_{i1} {\text{ I}}\left\{ {{\text{ t }} - {\text{ s}}_{i} > {\text{ c}}_{i} } \right\},$$

where I{ } is an indicator function equal to 1 if the time between the start of the lockdown and period t is greater than the changepoint c_*i*_. We use the segmented package in R to estimate the value of both of the slope parameters (β_*i*0_ and β_*i*1_) and the change point (c_*i*_) for each country *i*. For every country we compare the Akaike Information Criterion (AIC) of this model against a restricted model without a changepoint (i.e., β_*i*1_ = 0), selecting the specification with the smallest AIC for that country.

The selected model is used to assess whether each country exhibited a significant rebound in workplace mobility. This is achieved by verifying whether β_*i*0_ or β_*i*0_ + β_*i*1_ are significantly positive. The selected model is also used to estimate the number of days after reaching the minimum mobility during the lockdown (minwm_*i*_) it would take to return to the average mobility levels observed in the 2 weeks prior to the start of the lockdown (avgwm2wprior_*i*_). This is calculated for countries with significant mobility rebounds as follows:

- When the best fitting model does not have a changepoint and β_*i*0_ is significantly positive: (avgwm2wprior_*i*_ − minwm_*i*_)/β_*i0*_.
- When the best fitting model has a changepoint, β_*i*0_ is significantly positive, β_*i*0_ + β_*i*1_ is not significantly positive: (avgwm2wprior_*i*_ − minwm_*i*_)/β_*i*0_. In this case, we must verify whether this value is smaller than the country’s changepoint c_*i*_. If it is not smaller, then the estimated rebound does not reach the mobility levels prior to the lockdown and hence we don’t consider this value.
- When β_*i*0_ is not significantly positive, but β_*i*0_ + β_*i*1_ is significantly positive: c_*i*_ + (avgwm2wprior_*i*_ − minwm_*i*_)/(β_*i*0_ + β_*i*1_)
- When both β_*i*0_ and β_*i*0_ + β_*i*1_ are significantly positive: If (avgwm2wprior_*i*_ − minwm_*i*_)/β_*i*0_ > c_*i*_, then the days to return to the baseline level are calculated as
  c_*i*_ + (avgwm2wprior_*i*_ – estwmobc_i_)/(β_*i*0_ + β_*i*1_), otherwise this value is equal to (avgwm2wprior_*i*_ − minwm_*i*_)/β_*i*0_, where estwmobc_i_ is the predicted workplace mobility for country *i* at the regression changepoint c_i_.

We use country characteristics to explain differences in the mobility at the beginning of the lockdown, the fatigue or wear out, and the mobility drop observed at the beginning of the lockdown. We rely on the following country characteristics, with their respective sources in parenthesis:

i. *wm7before* 7-day moving average of the workplace mobility evaluated 7 days before the start of the lockdown (Google COVID-19 Community Mobility Reports).
ii. *si7before* 7-day moving average of the stringency index evaluated 7 days before the start of the lockdown (Oxford COVID-19 Government Response Tracker).
iii. *population* country population (Google COVID-19 Community Mobility Reports).
iv. *area* geographical area (squared kilometers, Google COVID-19 Community Mobility Reports).
v. *lifeexp* life expectancy (years, UNDP).
vi. *gnipc* gross national income per capita (UNDP).
vii. *expsch* expected years of schooling (UNDP).
viii. *meansch* mean years of schooling (UNDP).

In order to select the explanatory variables to use in each model, we rely on a stepwise regression with both addition and removal of explanatory variables in each step according to a p-value of 0.3 for entry and removal. We obtain identical conclusions if we use instead a p-value of 0.1 for inclusion and removal. The estimation is implemented in the R statistical computing and graphics software using the ols_step_both_p function. Detailed results are presented below.

The first model we estimate explains differences across countries in their mobility levels prior to the lockdown start, as measured by *wm7before*. Results are shown in Table 1.

**Table 1:** Estimation results for a linear model of mobility at the start of the lockdown (*wm7before*) as a function of country characteristics.

Dependent variable	*wm7before*		
Predictor	Estimate	t statistic	p-value
Intercept	17.99805	4.515	< 0.001
sibefore	− 0.50344	− 8.980	< 0.001
meansch	− 0.87645	− 2.378	0.020

R^2^: 0.580, Adjusted R^2^: 0.567; F-statistic: 44.85 on 2 and 65 degrees of freedom (n = 68).

The second model considers the reduction in mobility achieved during the lockdown as measured by the difference between avgwm2wprior_*i*_ and *minwm*_*i*_ which we denote as: *mobdropbefmin*_*i*_. Table 2 shows the estimated model.

**Table 2:** Estimation results for a linear model of the mobility reduction achieved during a lockdown as a function of country characteristics.

Dependent variable	*mobdropbefmin*		
Predictor	Estimate	t statistic	p-value
Intercept	34.438	1.746	0.086
avgsi7bfl	− 0.523	− 5.430	< 0.001
lifeexp	0.474	1.818	0.074

R^2^: 0.334, Adjusted R^2^: 0.313; F-statistic: 16.3 on 2 and 65 degrees of freedom (n = 68).

The third model considers the rebound in mobility as measured by the difference between the 7-day moving average workplace mobility observed 4 weeks after reaching the minimum mobility level and minwm_*i*_. We denote this measure by *wmrebound4weeks*_*i*_. We also include the reduction in mobility achieved between the start of the lockdown and the date when the minimum mobility is achieved (wmdropstartmin) as a regressor in this model. Table 3 shows the estimation results this model.

**Table 3:** Estimation results for a linear model of the mobility rebound as a function of country characteristics.

Dependent variable	*wmrebound4weeks*_*i*_		
Predictor	Estimate	t statistic	p-value
Intercept	12.308	5.949	< 0.001
wmdropstartmin	0.417	2.505	0.015
*wm7before*	− 0.220	− 2.353	0.022

R^2^: 0.148, Adjusted R^2^: 0.118; F-statistic: 4.86 on 2 and 56 degrees of freedom (n = 59).

## Supplementary Information

Supplementary Information 1.

Supplementary Information 2.

## Data Availability

The data that support the findings of this study are publicly available online at: Google: https://github.com/GoogleCloudPlatform/covid-19-open-data, Oxford: https://github.com/OxCGRT/covid-policy-tracker, UNDP: http://hdr.undp.org/sites/default/files/2020_statistical_annex_all.xlsx.

## Code availability

Software used includes R (https://cran.r-project.org/, version 4.1.1) and RStudio (https://www.rstudio.com/products/rstudio/download/, version 1.4.1717) for data analysis and model estimation. The code will be available from the authors upon request after publication (and for review).
